# Effect of Screen Time on Language Development Among Toddlers and Preschool Children in Al Qatif, Saudi Arabia

**DOI:** 10.7759/cureus.101696

**Published:** 2026-01-16

**Authors:** Faisal O AlQurashi, Hussain Alshammasi, Saleh Alhashim, Zahra Alrebh, Abdulraheem Almubarak

**Affiliations:** 1 Pediatrics, King Fahd Hospital of the University, Dammam, SAU; 2 Pediatrics, King Saud Bin Abdulaziz University for Health Sciences, Riyadh, SAU; 3 Pediatrics, Qatif Central Hospital, Al Qatif, SAU

**Keywords:** children, language development, parents, saudi arabia, screen time

## Abstract

Background

Speech and language development in early childhood is essential for cognitive and social development. With increasing exposure to screens at younger ages, concerns have been raised about potential effects on early language development. Although international recommendations advise limiting screen exposure, awareness and adherence remain variable.

Objectives

The primary objective is to examine the association between screen exposure patterns and communication scores on the Arabic Ages and Stages Questionnaire, third edition (A-ASQ‑3), among toddlers and preschool‑aged children in the Al Qatif region, Saudi Arabia.

The secondary objectives are to explore associations between children’s communication scores and maternal education, parental supervision, and familiarity with parental guidance (PG) content ratings during screen use, and children’s sleep duration and related sleep habits.

Methods

A cross‑sectional community‑based study with prospective data collection was conducted between February 2023 and July 2024. Children aged 1-66 months were included because the A-ASQ‑3 provides standardized age‑specific forms across this range, allowing consistent assessment of the communication domain in infants, toddlers, and preschool‑aged children. Data were collected using a parent‑completed questionnaire and analyzed using the IBM SPSS Statistics for Windows, Version 29 (Released 2022; IBM Corp., Armonk, New York, United States). The primary outcome was language development status assessed using the A-ASQ‑3 Communication domain (Arabic version). According to A-ASQ‑3 scoring guidelines, children were categorized as above the cut‑off, close to the cut‑off, or below the cut‑off. For interpretation, "language delay" referred to scores below the cut‑off on the communication domain.

Results

Among 98 children, 35 (36.1%) were introduced to electronic devices between one and two years of age. Daily screen use was most commonly one to two hours/day (33 children, 33.7%), followed by two to three hours/day (23 children, 23.5%). Thirty‑six parents (36.7%) reported implementing screen‑time restrictions. Televisions and smartphones were the most frequently available devices, and 74 children (75.5%) mainly watched children’s entertainment shows. Higher language development scores were significantly associated with higher maternal education, caregiver familiarity with PG scores, children sleeping more than 10 hours per night, and parental supervision during screen use, whereas daily screen time duration alone was not significantly associated with scores. The prevalence of delayed language development (scores below the A-ASQ‑3 communication cut‑off) was 6.1%.

Conclusion

In this cross‑sectional study, language development scores were more favourable among children whose mothers had higher education levels, whose caregivers were familiar with content guidance ratings, who slept longer, and who received closer parental supervision during screen use. These findings support family counselling that emphasizes supervised screen use, age‑appropriate content, and healthy sleep routines, while raising awareness of language development milestones. Larger, longitudinal studies are required to clarify directionality and long‑term outcomes.

## Introduction

Speech and language development play a critical role in reflecting the broader cognitive abilities and overall development of a child [1]. Speech refers to verbal production, whereas language (receptive or expressive) encompasses human communication, enabling the sharing of emotions, ideas, information, and beliefs. Receptive language involves understanding, while expressive language pertains to conveying information and feelings [2]. Screen time, as defined in current literature, refers to the time spent in front of a screen, including phones, video games, televisions, computers, laptops, and tablets. This term encompasses both active and passive screen time [3]. Passive screen time involves inactive screen-based activities or passive absorption of digital information, whereas active screen time refers to the intellectual or physical engagement of a child in digital activities. Differentiating between these types is essential to understanding their distinct developmental impacts [4].

Early childhood is a critical period for rapid brain, cognitive, and language development, during which foundational speech and language skills are acquired and essential for learning and social interaction [2,5]. During this sensitive period, as language develops rapidly with the aid of environmental stimulation and linguistic input from caregivers, screen exposure plays a crucial role [6]. Screen use in various forms has become increasingly prevalent among children, as these technologies are now embedded in our daily lives. Children’s experiences are increasingly shaped by an ever-evolving digital ecosystem that is continually enhanced through interactive mobile media [7].

Recent research has shown a notable decline in the age of first digital media exposure, with television use beginning around eight months and smartphone interaction soon after [8]. According to the WHO guidelines, screen time is not recommended for one-year-old infants, and for children aged two to four years, it should be limited to no more than one hour per day of sedentary screen time [9]. The UK guidelines established by the National Institute for Clinical Excellence recommend limiting leisure screen time to two hours daily for all children. The American Academy of Pediatrics (AAP) cautions against screen exposure before 18 months of age, excessive screen time for children aged two to five years, and strict restrictions for older children. Despite the international adoption of these principles, awareness and compliance remain inadequate [10].

In a meta-analysis of 95 international studies conducted in 2020 involving 89,163 children, 24.7% of children aged less than two years adhered to the AAP recommendations to limit screen use. In addition, 35.6% of children aged two to five years followed the AAP guideline of limiting screen time to one hour per day. The same study noted an increase in compliance with these guidelines in recent years [11]. A 2017 national cross‑sectional survey report from the United States found that 71% of children began watching television before age two, and 46% used mobile media before that age. The report also indicated that 98% of children aged up to eight years lived in homes with internet‑connected devices and spent, on average, more than two hours per day using screens, with a mean daily screen time of 42 minutes for children aged up to two years and 2.39 hours for those aged two to four years [12]. Similarly, a 2018 study conducted in Saudi Arabia showed that 94% of children started watching TV, and 79% started using mobile media before age two. The average screen time for children aged one to three years is 3.14 hours per day [5].

Several global and local studies explored the link between childhood development (including language) and screen time. A 2020 meta-analysis of 223 studies revealed that excessive screen time (over two hours per day) among infants, toddlers, and preschoolers was associated with diverse health indicators across physical, behavioral, and psychosocial domains [13]. A 2013 study conducted in the US focused on 119 Hispanic infants and toddlers and reported that excessive TV viewing (over two hours per day) was linked to lower communication scores. Exposure to child-directed media correlated with poor language scores, whereas adult-directed media showed no such effect [14]. In 2019, a South Korean cross-sectional study reported a negative correlation between screen (primarily mobile devices) usage time and expressive language skills among three-year-olds [15]. Similarly, a 2023 cross-sectional study in the Philippines revealed that spending more than two hours on screens was significantly linked to a decline in both receptive and expressive language scores [16].

Several local studies related to this topic have been identified. A 2017 cross-sectional descriptive study conducted in the eastern province of Saudi Arabia assessed the language development of children aged three to five years using the Arabic Ages and Stages Questionnaire, third edition (A-ASQ-3) and parental reports on social interactions. The findings indicate that language delay is associated with watching TV for over two hours per day and spending less than two hours daily with their mothers [17]. Similarly, a survey-based cross-sectional study conducted in 2020 in Riyadh, Saudi Arabia, revealed a significant association between spending more than two hours on mobile devices and language development [18]. Finally, a 2018 cross-sectional study conducted in Saudi Arabia reported that screen media quantity was negatively correlated with the words produced among children aged 17-36 months [5].

In contrast, some positive aspects include keeping children active while ensuring their safety, enhancing vocabulary, exposing them to various activities, and promoting cultural and linguistic diversity [5,19]. The development of speech and linguistic, physical, cognitive, and social skills has favorable benefits, although negative consequences have been observed. Children begin to grasp information after the age of two, and they may struggle to apply the knowledge acquired through the screen of a device [5,18,20].

In Saudi Arabia, studies on children aged less than three years are scarce, and the beneficial and harmful effects of using electronics and screen time remain unclear. This study examined the association between screen‑time exposure and language development in children aged 1-66 months in Al Qatif, Saudi Arabia, and explored sociodemographic and media‑context factors associated with language scores. Despite increasing screen exposure among young children, evidence from Saudi Arabia, particularly from the Eastern Province, remains limited regarding how screen time patterns (duration, supervision, content type, and timing, including screen use close to bedtime) relate to early language development. Local data are needed to inform caregiver counselling and public health guidance.

## Materials and methods

Study design

This descriptive, cross‑sectional community‑based study with prospective data collection at a single time point included children aged 1-66 months residing in the Al Qatif region, a governorate and urban area located in the Eastern Province of Saudi Arabia, between February 2023 and July 2024.

Study population and sampling

The inclusion criteria were toddlers and preschoolers aged 1-66 months, corresponding to the age range covered by the standardized A-ASQ-3 communication domain forms and encompassing infancy, toddlerhood, and preschool years, of both sexes, living in the Al Qatif region, and of Saudi nationality. The exclusion criteria were children diagnosed with hearing impairment, mental retardation, or other metabolic or genetic disorders affecting development; guardians refusing to participate in the study; and incomplete surveys.

**Figure 1:**
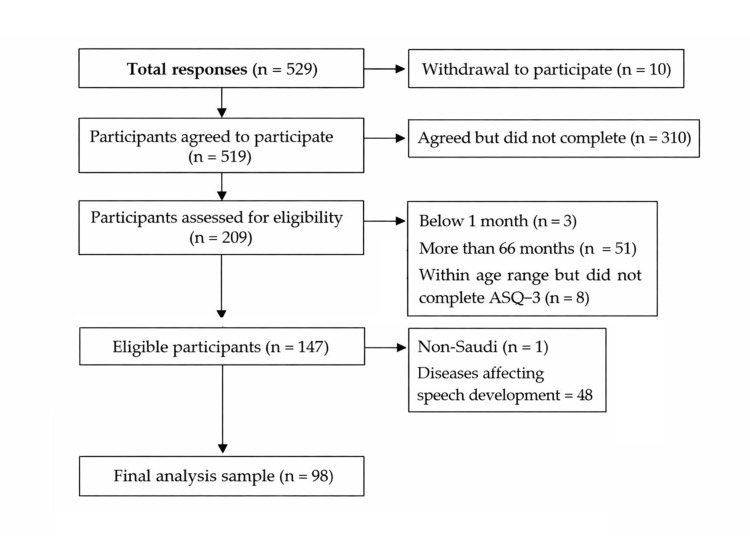
Selection process of the study sample ASQ-3: The Ages and Stages Questionnaire, third edition

A non‑random convenience sampling technique was used. An a priori sample size of 377 participants was estimated using the Raosoft calculator (Raosoft Inc., Seattle, WA, USA) (5% significance level, 5% margin of error, 95% confidence level, and an assumed response distribution of 50%) [21]. However, after exclusions for ineligibility and incomplete A-ASQ‑3 data, the final analyzed sample comprised 98 children, as illustrated in Figure 1.

Data collection tool and technique

Data were collected using a parent‑completed, self‑administered questionnaire (primarily distributed online). Trained data collectors were available to clarify questions when needed. The questionnaire consisted of four main parts: children’s demographic data, parents’ demographic characteristics, screen exposure and sleep‑related variables, and language development scores (see Appendices).

Parents’ demographic data included age, occupation, education level, socioeconomic status, marital status, and number of children in the family. Household variables included whether the child lived only with parents and siblings or with an extended family. "Living with extended family" was defined as the child residing in a household that includes relatives beyond parents and siblings (e.g., grandparents, uncles, aunts, or cousins). Children’s data (for each toddler and preschooler in the family) included age, sex, any chronic diseases, and basic media‑use characteristics (e.g., whether the child used their own or shared devices and whether viewing usually occurred with supervision). Child age was recorded in predefined categorical bands (e.g., 1-3, 3-5, 5-7 months … 57-66 months) in accordance with the A-ASQ‑3 age intervals and is presented as categorical data in the results.

Screen exposure variables were defined a priori as follows: daily screen‑time duration (none, <30 minutes, one to two hours, two to three hours, three to four hours, more than four hours), age at introduction of electronic devices, number and type of devices in the household, parental restriction of screen time, parental supervision during screen use, use of screens as a means of calming or distraction, presence of background media, and screen use during the last two hours before sleep. Sleep‑related variables included child sleep problems (yes/no) and total sleep duration per day (5-9, 10-14, >14 hours).

For the purposes of analysis, these screen exposure and sleep variables were treated as independent (predictor) variables. The dependent variable (presumed effect) was language development status, assessed using the A‑ASQ‑3 communication domain, scored according to standard age‑specific guidelines [22].

Data analysis

Data were analyzed using the IBM SPSS Statistics for Windows, Version 29 (Released 2022; IBM Corp., Armonk, New York, United States). For categorical data, descriptive statistics were calculated as frequencies and percentages, whereas continuous variables were calculated as measures of central tendency (arithmetic mean and median) and dispersion (standard deviation and range). The results are provided in tables and figures. The chi-square test was used to determine the relationship among language development score, screen time, and parents’ sociodemographic characteristics. A p-value ≤ 0.05 was considered a significant relationship.

Ethical consideration

The investigators obtained written informed consent from all participating caregivers, and each had the right to accept or refuse participation and to withdraw at any time. Participants were informed about the study objectives and the precautions taken to protect data confidentiality. Security procedures (such as encryption and password protection) were applied during data transfer to SPSS, and no identifying information (such as names, telephone numbers, or national IDs) was collected. This study was approved by the Institutional Review Board of Qatif Central Hospital, Al Qatif, Saudi Arabia (QCH‑SRECO 23/2024), on 3 June 2024, before initiation of data collection.

## Results

Table 1 shows that the study examined 98 children aged 1-66 months. The age group with the highest frequency was 57-66 months, comprising 15 (15.3%) children, followed closely by those aged 34.5-39 months (13, 13.3%). Most of the participants were male (51, 52%). Moreover, 63 children (63.3%) did not attend kindergarten, and only 12 (12.2%) had a helper or nanny at home. Fifty-eight children (58.2%) lived with extended families, and 60 (60.2%) came from families with two or three children. In addition, the sample displayed varied distribution in birth orders, with firstborns accounting for 31 (31.6%) children and thirdborns comprising 27 (27.6%) children.

**Table 1:** Demographic characteristics of children (n = 98)

Demographic characteristics		Frequency	Percentage (%)
Age	1-3 months	1	1.0
	3-5 months	1	1.0
	5-7 months	2	2.0
	7-9 months	5	5.1
	9-10 months	1	1.0
	10-11 months	1	1.0
	11-13 months	1	1.0
	13-15 months	2	2.0
	15-16 months	1	1.0
	17-19 months	2	2.0
	19-21 months	2	2.0
	21-23 months	6	6.1
	23-25.5 months	10	10.2
	25.5-28.5 months	2	2.0
	28.5-31.5 months	6	6.1
	31.5-34.5 months	5	5.1
	34.5-39 months	13	13.3
	39-45 months	9	9.2
	45-51 months	9	9.2
	51-57 months	4	4.1
	57-66 months	15	15.3
Sex	Female	47	48.0
	Male	51	52.0
Attend kindergarten	Yes	36	36.7
	No	62	63.3
Nanny presence at home	Yes	12	12.2
	No	86	87.8
Living with extended family	Yes	57	58.2
	No	41	41.8
Number of children in the family	1 child	29	29.6
	2-3 children	59	60.2
	4-5 children	8	8.2
	More than 5 children	2	2.0
Birth order	1.00	31	31.6
	2.00	25	25.5
	3.00	27	27.6
	4.00	5	5.1
	5.00	2	2.0
	6.00	1	1.0
	7.00	7	7.1

Table 2 shows that most of the informants (57, 58.2%) were mothers, approximately 48 families had both parents employed (49.0%), while 50 relied solely on the income of the father (51.0%). Regarding parental education, 68 (69.4%) held a diploma/bachelor's degree, 17 (17.3%) had a high school education, and 12 held a master’s/PhD degree (12, 12.2%). Of mothers, 78 (79.6%) held a diploma/bachelor's degree, 11 had a high school education (11.2%), and seven had a Master’s/PhD (7.1%). Regarding household income, the most common range was 10,001-20,000 SAR (45 households, 45.9%), followed by those exceeding 20,000 SAR (19, 19.4%) and 5,001-10,000 SAR (25, 25.5%). The primary language spoken in most households was Arabic (82, 83.7%), with a smaller proportion using Arabic and English (16, 16.3%). Finally, 62 households engaged in interactive communication for more than two hours daily (63.3%), while 26 interacted for one to two hours (26.5%), and 10 interacted for less than one hour (10.2%).

**Table 2:** Parent’s demographic characteristics (n = 98) PhD: Doctor of Philosophy; SAR: Saudi Riyal

Demographic characteristics		Frequency	Percentage (%)
Informant	Father	38	38.8
	Mother	57	58.2
	Others	3	3.1
Marital status	Married	98	100.0
	Divorced	0	0
Employment status	Both parents employed	48	49.0
	Father only	50	51.0
Father’s educational	Less than high school	1	1.0
	High school	17	17.3
	Diploma/Bachelor	68	69.4
	Master/PhD	12	12.2
Mother’s education	Less than high school	2	2.0
	High school	11	11.2
	Diploma/Bachelor	78	79.6
	Master/PhD	7	7.1
Monthly income (SAR)	<5000	9	9.2
	5001-10,000	25	25.5
	10,001-20,000	45	45.9
	>20,000	19	19.4
Language spoken	Arabic	82	83.7
	Mix (Arabic and English)	16	16.3
Interactive communication	<1 hour/day	10	10.2
	1-2 hours/day	26	26.5
	>2 hours/day	62	63.3

Table 3 summarizes children’s screen exposure and sleep patterns. Thirty‑five caregivers (35.7%) reported introducing electronic devices to their children between the ages of one and two years, and 20 (20.4%) reported introduction between two and three years. Very early exposure before six months was uncommon (two children, 2.0%), whereas 13 children (13.3%) were not allowed to use electronic devices. Most households had multiple screen devices; 43 caregivers (43.9%) reported three to four devices at home, and 21 (21.4%) indicated that their child owned a device. Daily screen time was most often one to two hours per day (33 children, 33.7%) or two to three hours per day (23 children, 23.5%), with a smaller group (six children, 6.1%) exceeding four hours per day. These categories correspond to commonly used clinical thresholds for counselling caregivers but represent broad time intervals based on parental report.

**Table 3:** Screen time exposure and sleep pattern PG: parental guidance

Variables		Frequency	Percentage (%)
Age of introduction of electronic devices	<6 months	2	2.0
	6-12 months	13	13.3
	Between 1 and 2 years	35	35.7
	Between 2 and 3 years	20	20.4
	>3 years	15	15.3
	Not allowed	13	13.3
Number of screen devices at home	None	3	3.1
	1-2 devices	23	23.5
	3-4 devices	43	43.9
	5-6 devices	18	18.4
	>6	11	11.2
Does your child own a device	No	77	78.6
	Yes	21	21.4
Daily duration of screen time	None	10	10.2
	<30 min	21	21.4
	1-2 hours/day	33	33.7
	2-3 hours/day	23	23.5
	3-4 hours/day	5	5.1
	>4 hours	6	6.1
Following the PG score for movies and video games	Yes	49	50.0
	No	26	26.5
	Not familiar with PG	23	23.5
Restriction on-screen device time	No	62	63.3
	Yes	36	36.7
Duration of restriction	No restriction	62	63.3
	1 hour/day	14	14.3
	2 hours/day	13	13.3
	3 hours/day	7	7.1
	4 hours/day	2	2.0
Screen exposure during the last two hours before sleep	Yes	42	42.9
	No	56	57.1
Child sleep problems	Yes	12	12.2
	No	86	87.8
Total sleep duration per day (hours)	5-9 hours	28	28.6
	10-14 hours	67	68.4
	>14 hours	3	3.1

Regarding parental management of media, 36 caregivers (36.7%) reported implementing explicit limits on screen time, most frequently one or two hours per day, and 42 parents (42.9%) reported that their child was exposed to screens within the last two hours before sleep. Familiarity with parental guidance (PG) scores for movies and video games varied: 49 caregivers (50.0%) reported following PG scores, 26 (26.5%) did not follow them, and 22 (23.5%) were unfamiliar with these ratings. In this study, PG familiarity was used as an indicator of caregivers’ awareness of age‑appropriate content classifications. With respect to sleep, 67 children (68.4%) were reported to sleep 10-14 hours per day, whereas 28 (28.6%) slept five to nine hours, and 12 (12.2%) were described as having sleep problems. These descriptive patterns provide the basis for examining how screen exposure, supervision, PG familiarity, and sleep relate to children’s language development scores, while recognizing that the exposure measures rely on caregiver report and broad duration categories.

**Figure 2:**
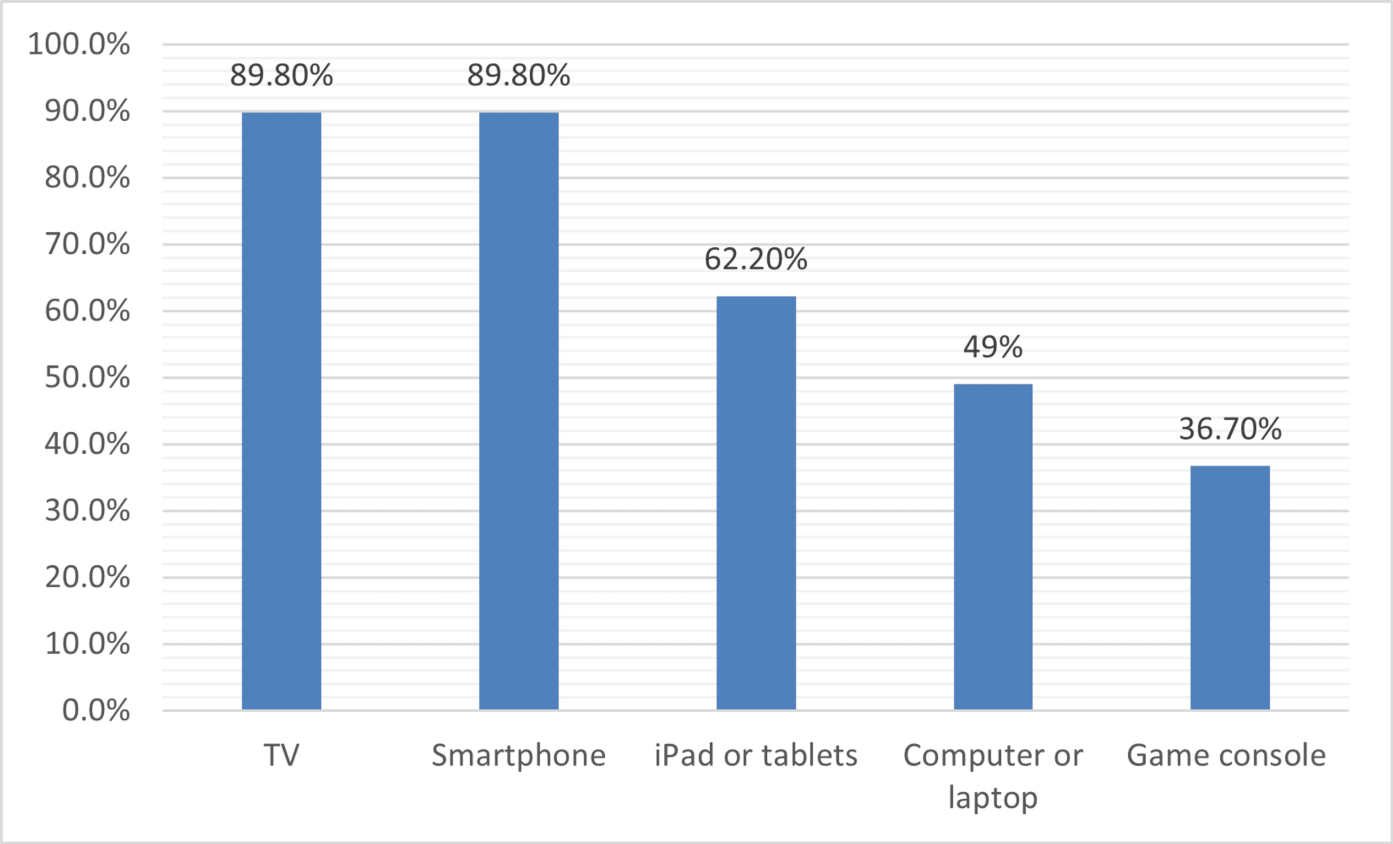
Types of screen media devices available at home Game console (e.g., PlayStation, Xbox)

Figure 2 shows that most families had TVs (88, 89.8%) and smartphones (88, 89.8%), iPads or tablets (61, 62.2%), computers or laptops (48, 49.0%), and 36 (36.7%) had gaming consoles.

**Figure 3:**
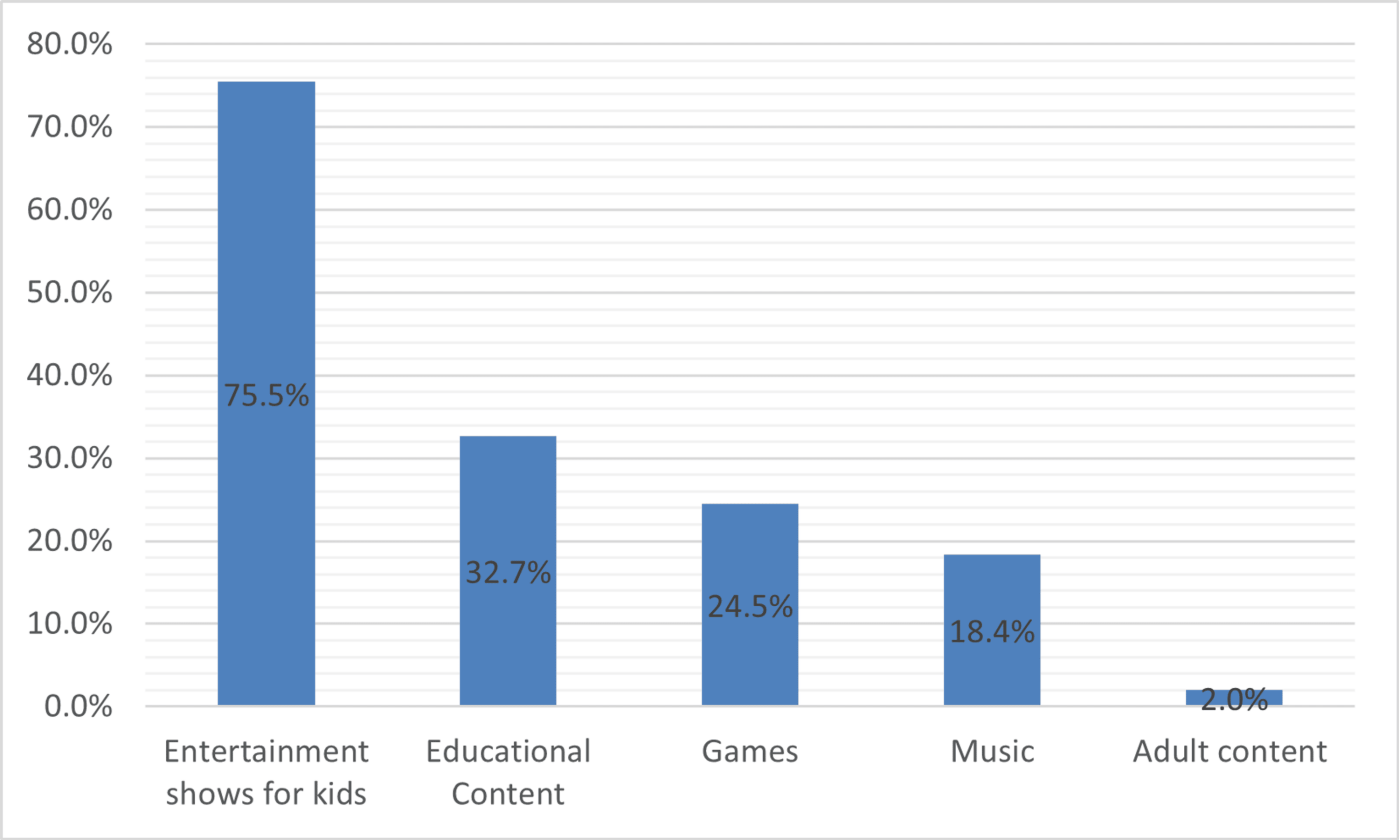
Nature and language of content Entertainment shows for kids include cartoons and animated programs; Educational content includes videos, puzzles, reading, and math games; Adult content includes movies or TV shows primarily intended for adults.

Figure 3 shows that 74 families (75.5%) reported that their children primarily watched entertainment shows designed for children. Educational content (videos, puzzles, reading, and math games) was popular (32, 32.7%). This was followed by games (24, 24.5%) and music (18, 18.4%). Exposure to adult content was minimal, affecting only two children (2, 2%).

**Figure 4:**
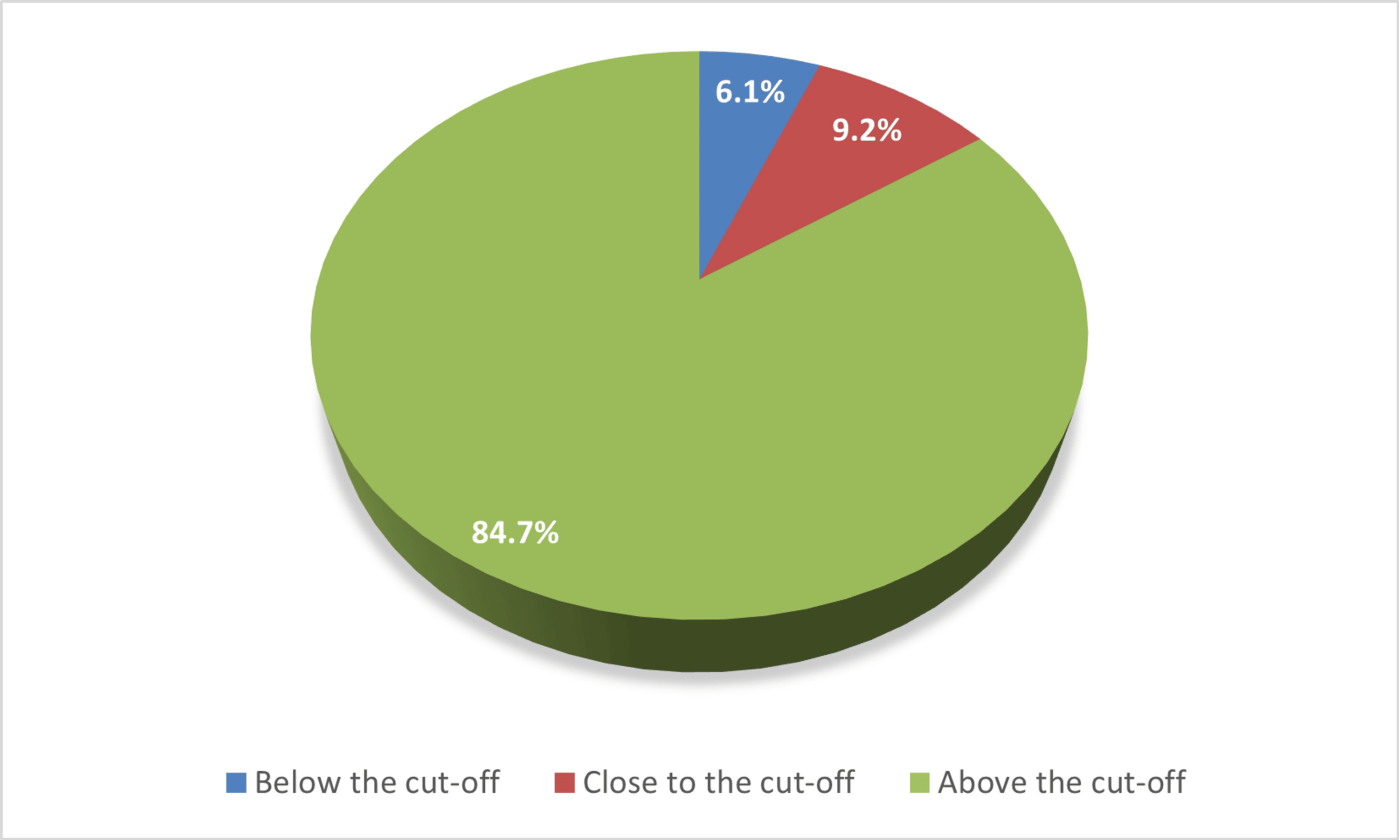
Children’s language development scores assessed by the A-ASQ-3 A-ASQ: Arabic Ages and Stages Questionnaire, Third Edition

Figure 4 reveals that 83 (84.70%) children scored above the cutoff on the A-ASQ-3, indicating good language development. In contrast, six (6.10%) children scored below the cutoff on the A-ASQ-3, indicating potential delays in language development. In addition, nine children had (9.2%) scores close to the cutoff, which may warrant further evaluation to identify any potential areas of development.

**Figure 5:**
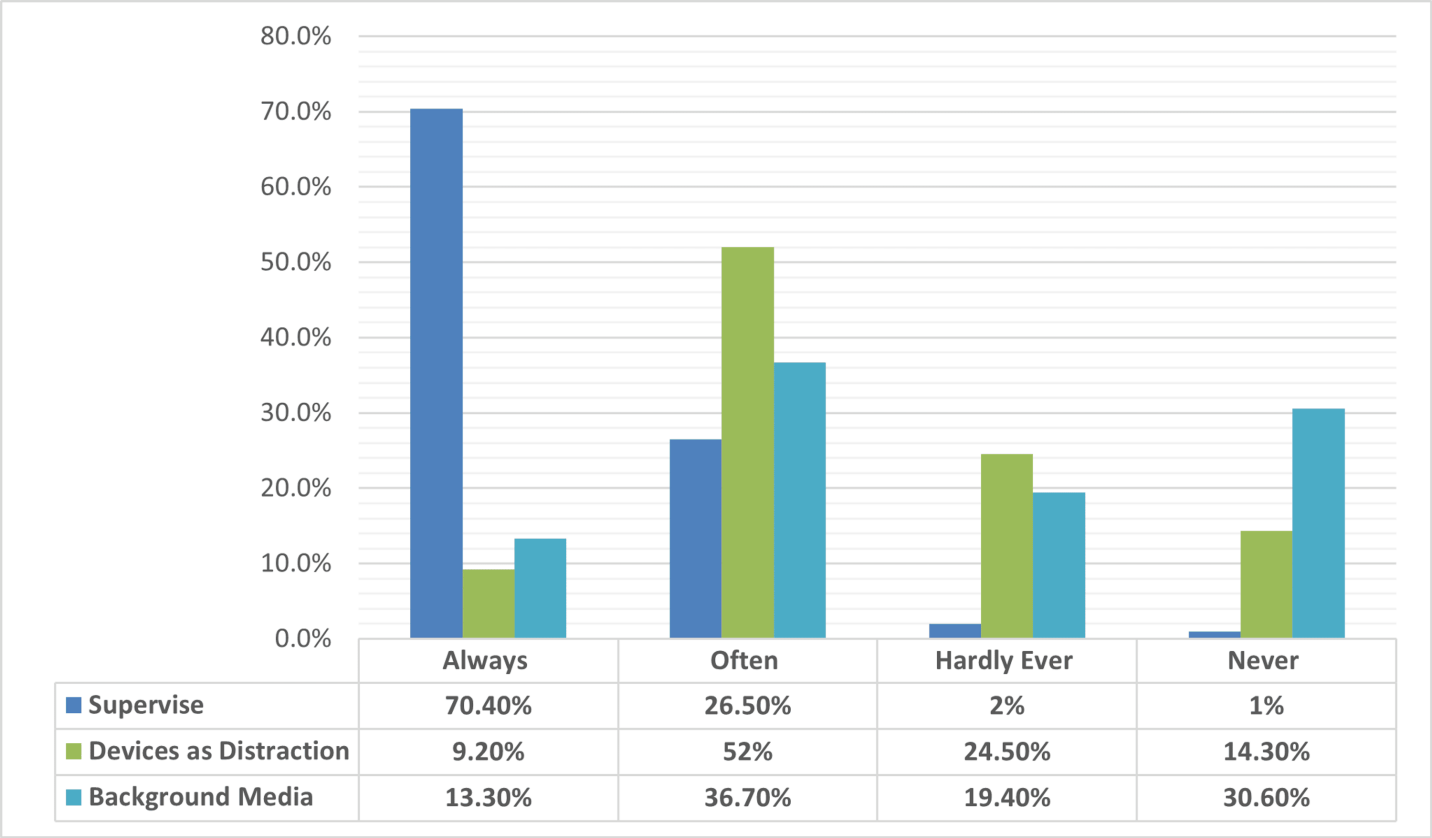
Parental supervision, use of devices as a distraction, and background media exposure

Figure 5 reveals that 69 parents (70.4%) always supervised their children during screen time. Fifty-one (52.0%) often used screens to calm or distract their children, while 36 (36.7%) often had screens in the background at home (such as during meal time). Conversely, 30 parents (30.6%) reported never having background screen use.

Table 4 indicates a significant relationship between the language development scores of the informants and children, with the mother being associated with a higher score. Thus, the informants reporting on language development may have influenced the results. No statistically significant difference was observed in language development scores based on the employment status of parents and paternal education. The educational level of the mothers showed a statistically significant association with the language development scores of their children. Children whose mothers had less than a high school education scored lower on the A-ASQ-3 than those whose mothers had higher education levels.

**Table 4:** Relationship between parent’s characteristics and children’s language development scores * Significant at P < 0.05.

Parent’s characteristics		Children’s language development score						P-value
		Below the cutoff		Close to the cutoff		Above the cutoff		
		N	%	N	%	N	%	
Informant	Father	2	5.3	6	15.8	30	78.9	0.029*
	Mother	3	5.3	2	3.5	52	91.2	
	Others	1	33.3	1	33.3	1	33.3	
Employment	Both parents employed	2	4.2	4	8.3	42	87.5	0.688
	Father only	4	8.0	5	10.0	41	82.0	
Father’s education	Less than high school	0	0.0	0	0.0	1	100	0.916
	High school	1	5.9	3	17.6	13	76.5	
	Diploma/Bachelor	4	5.9	5	7.4	59	86.8	
	Master/PhD	1	8.3	1	8.3	10	83.3	
Mother’s education	Less than high school	0	0.0	2	100	0	0.0	0.001*
	High school	1	9.1	0	0.0	10	90.9	
	Diploma/Bachelor	5	6.4	6	7.7	67	85.9	
	Master/PhD	0	0.0	1	14.3	6	85.7	

Table 5 reveals that no statistically significant difference was observed in language development scores based on the daily duration and restrictions on screen time. Children whose parents were familiar with the PG scores for movies and video games had significantly higher language development scores than those without this guidance (P = 0.003).

**Table 5:** Relationship between screen time characteristics and children’s language development scores * Significant at P < 0.05.

Variables		Children’s language development score						P-value
		Below the cutoff		Close to the cutoff		Above the cutoff		
		N	%	N	%	N	%	
Daily duration of screen time	None	0	0	3	30.0	7	70.0	0.487
	<30 minutes	1	4.8	2	9.5	18	85.7	
	1-2 hours/day	2	6.1	2	6.1	29	87.9	
	2-3 hours/day	2	8.7	1	4.3	20	87.0	
	3-4 hours/day	0	0	0	0	5	100.0	
	>4 hours	1	16.7	1	16.7	4	66.7	
Following the parental guidance (PG) score for movies and video games	Yes	2	4.1	1	2.0	46	93.9	0.003*
	No	0	0	6	23.1	20	76.9	
	Not familiar with PG	4	17.4	2	8.7	17	73.9	
Restriction on screen device time for your child	No	4	6.5	4	6.5	54	87.1	0.468
	Yes	2	5.6	5	13.9	29	80.6	
Age of introduction	<6 months	0	0.0	0	0	2	100	0.614
	6-12 months	2	15.4	1	7.7	10	76.9	
	Between 1 and 2 years	2	5.7	4	11.4	29	82.9	
	Between 2 and 3 years	1	5.0	1	5.0	18	90.0	
	>3 years	1	6.7	0	0	14	93.3	
	Electronic devices not allowed for my child	0	0	3	23.1	10	76.9	
Interactive communication	<1 hour/day	1	10.0	0	0	9	90.0	0.449
	1-2 hours/day	3	11.5	3	11.5	20	76.9	
	>2 hours/day	2	3.2	6	9.7	54	87.1	

Table 6 shows that no significant relationship was observed between screen exposure within two hours of sleep and language development scores. Children who experience sleeping problems are more likely to score below the cutoff for language development compared to those without sleeping problems. However, this difference was not statistically significant. Furthermore, children who sleep <10 hours per night showed lower language development scores than those who sleep >10 hours. This difference was statistically significant (P = 0.012), suggesting that adequate sleep is crucial for optimal language development.

**Table 6:** Relationship between sleep and children’s language development scores * Significant at P < 0.05.

Variables		Children’s language development score						P-value
		Below the cutoff		Close to the cutoff		Above the cutoff		
		N	%	N	%	N	%	
Any screen exposure during the last two hours before sleep?	Yes	3	7.1	3	7.1	36	85.7	0.792
	No	3	5.4	6	10.7	47	83.9	
Does your child experience sleeping problems?	Yes	2	16.7	0	0	10	83.3	0.154.
	No	4	4.7	9	10.5	73	84.9	
How many hours of sleep does your child have?	5-9 hours	2	7.1	3	10.7	23	82.1	0.012*
	10-14 hours	4	6.0	4	6.0	59	88.1	
	>14 hours	0	0	2	66.7	1	33.3	

Table 7 indicates that no clear significant correlation was observed between children's language development scores and the use of TV or mobile devices for calming or distracting purposes. However, a significant relationship was observed between parental supervision during children's screen time and language development (P = 0.008). Children whose parents supervised screen time were more likely to have higher language development scores (87.0%) than those whose parents provided less frequent supervision. In addition, the presence of TV or mobile media playing in the background at home has an impact. A higher percentage of children with above-the-cutoff language development scores reported "never" having background media at home during meal times (P = 0.036).

**Table 7:** Relationship between family behavior regarding screen time and children’s language development scores * Significant at P < 0.05.

Family’s media usage		Children’s language development score						P-value
		Below the cutoff		Close to the cutoff		Above the cutoff		
		N	%	N	%	N	%	
Do you supervise your child during active time on screen or mobile devices?	Always	4	5.8	5	7.2	60	87.0	0.008*
	Often	1	3.8	3	11.5	22	84.6	
	Hardly ever	1	50.0	0	0	1	50.0	
	Never	0	0	1	100	0	0	
Do you use TV or mobile devices to calm your child or as a means of distraction?	Always	2	22.2	0	0	7	77.8	0.389
	Often	3	5.9	6	11.8	42	82.4	
	Hardly ever	1	4.2	2	8.3	21	87.5	
	Never	0	0	1	7.1	13	92.9	
Do you have TV or mobile media playing in the background at home (for example, during mealtime)	Always	3	23.1	0	0	10	76.9	0.036*
	Often	2	5.6	4	11.1	30	83.3	
	Hardly ever	0	0	4	21.1	15	78.9	
	Never	1	3.3	1	3.3	28	93.3	

Table 8 reveals that screen time duration varies based on the patterns of content consumption, with statistically significant associations observed for games (P = 0.025) and entertainment shows for children (P < 0.001). These associations highlight the need to consider the nature and language of content within the broader context of children’s overall media use and its potential impact on development.

**Table 8:** Relationship between the nature and language of content and daily duration of screen time * Significant at P < 0.05.

Nature and language of content	Daily duration of screen time												P-value
	None		<30 minutes		1-2 hours/day		2-3 hours/day		3-4 hours/day		>4 hours		
	N	%	N	%	N	%	N	%	N	%	N	%	
Educational	4	12.5	6	18.8	9	28.1	10	31.3	0	0	3	9.4	0.379
Games	0	0	2	8.3	9	37.5	7	29.2	2	8.3	4	16.7	0.025*
Entertainment shows for kids	3	4.1	12	16.2	30	40.5	19	25.7	5	6.8	5	6.8	<0.0001
Music	0	0	8	44.4	5	27.8	3	16.7	0	0	2	11.1	0.065
Adult content	1	50.0	1	50.0	0	0	0	0	0	0	0	0	0.376

## Discussion

This descriptive cross‑sectional community‑based study conducted in the Eastern Province of Saudi Arabia examined the association between screen exposure patterns and language development scores among children aged 1-66 months, using the A-ASQ‑3 communication domain. Children in this sample were commonly introduced to electronic devices in the second and third years of life, had access to multiple screen devices at home, and typically used screens for one to three hours per day. Higher communication scores were associated with higher maternal education, closer parental supervision during screen use, longer sleep duration, and caregiver familiarity with PG content ratings, whereas daily screen time duration alone was not significantly related to language scores. In the following sections, we contextualize these findings by discussing screen exposure patterns, the role of parental supervision and content guidance, and related factors such as device availability, content type, and sleep, while also comparing our results with prior studies and highlighting key strengths and limitations.

Screen exposure patterns and supervision

The age of introduction and typical duration of daily screen exposure observed in this study are broadly comparable to those reported in regional and international studies. A previous study conducted in the UAE revealed that the early onset of electronic device use occurred at 12-24 months of age, which is consistent with our results [6]. Studies from different regions of Saudi Arabia have shown that parents frequently use digital media to keep children calm or occupied [23], and that a sizeable proportion of preschool children are exposed to screens for two to three hours per day [24], patterns that align with the present findings. A study in Jeddah, Saudi Arabia, reported a mean screen‑device usage of 3.1 hours per day (SD = 2.58) [25]. In India, a study reported that the mean screen time was 2.39 hours per day [26]. At the same time, larger cohorts, including a cross‑sectional study of more than 3,000 children in Brazil, have found that excessive screen time increased with age and was inversely associated with communication skills, with each additional hour linked to reduced communicative ability [27]. Taken together, these findings suggest that screen exposure has become normative in early childhood, but the strength and direction of its association with language outcomes may depend on the broader family and media context. These exposure patterns overlap with, and in some cases may exceed, conservative international recommendations that discourage screen use before 18-24 months and advocate limited, high‑quality content thereafter, and they should therefore be interpreted in light of our self‑reported, broad exposure measures and modest sample size.

In the current study, parental supervision during children's active screen use and familiarity with PG content ratings were positively associated with communication scores. Supervision was measured as the caregiver‑reported frequency of actively supervising the child while using screens, and PG familiarity captured caregivers’ awareness and reported adherence to age‑based content classifications for movies and video games; these variables are therefore best interpreted as general indicators of parental monitoring and attention to media appropriateness rather than detailed measures of interactive co‑viewing. In our sample, 36.7% of children were reported to receive parental supervision during screen use, compared with 57.8% in a previous study from Jeddah, Saudi Arabia [25], which may reflect differences in measurement or population context. The observed associations are correlational and consistent with developmental frameworks that emphasize the importance of contingent caregiver interaction and moderated exposure; however, given the cross‑sectional design, potential residual confounding, and reliance on parental self‑report, supervision, and PG familiarity cannot be considered independently protective. In light of these observations and existing guideline recommendations, potential family‑level strategies include establishing consistent screen‑time limits, minimizing background media, avoiding screen use in the hours before bedtime, and encouraging caregivers to select age‑appropriate content and remain engaged during children’s screen use, while future longitudinal studies clarify whether these practices directly improve language outcomes or primarily reflect broader caregiving patterns.

Device availability, content patterns, and sleep

Most participating households owned multiple devices. The percentages of families who owned televisions, smartphones, iPads or tablets, computers or laptops, and gaming consoles were 89.8%, 89.8%, 62.2%, 49.0%, and 36.7%, respectively, which were higher than those reported in some previous studies. In Jeddah, Saudi Arabia, the most used smart device was reported as a tablet (47%) [25], and smartphones were used by 42.4% of children [24]. Device ownership has been linked to increased technology use in several observational studies and literature reviews [28]. Regional differences in socioeconomic development, cultural norms, and family policies likely contribute to variability in device availability across studies. While these data demonstrate that most households own several types of screen devices, our results describe availability rather than implying that each device contributes equally to developmental risk. The study did not differentiate exposure by device modality or examine dose-response relationships between device counts and language outcomes; therefore, interpretations should focus on the broader media environment rather than specific devices.

Our results align with those of previous studies in Saudi Arabia, which reported entertainment (60.8%) as the most consumed content, followed by games (47.6%) [25] and cartoons (42%) [24]. The alignment of the current findings with these earlier studies supports the generalizability of content consumption patterns within the region, suggesting that cultural and societal factors influence children's media preferences. Although the present study did not differentiate language outcomes by specific content types or platforms, this consistency in content preferences is noteworthy. Future studies should explore the long‑term effects of different types of content on children's development and well‑being.

Regarding sleep, a majority of children were reported to sleep 10-14 hours per day (68.4%), but nearly one‑third slept five to nine hours (28.6%), and sleep problems were relatively common (12.2%). Children who slept longer (≥10 hours per day) had higher communication scores, in line with previous studies linking adequate sleep duration to more favourable cognitive and language outcomes. In contrast, screen exposure during the two hours before bedtime was not significantly associated with communication scores in this sample, although a substantial proportion of parents (42.9%) reported late‑evening screen exposure. Given the cross‑sectional design and modest sample size, these findings should be interpreted cautiously; nonetheless, they underscore the importance of considering sleep as part of the broader media environment when evaluating early language development.

Comparison with previous studies

The prevalence of delayed language development (6.1%) in this study is similar to estimates from some Saudi cohorts but differs from others. A previous study in Makkah, Saudi Arabia, reported a 5.6% prevalence of developmental communication delay [29], whereas a cross‑sectional study at three primary healthcare centers in Riyadh, Saudi Arabia, reported that language delay was more prominent in the one‑year‑old age group (26.7%) [30]. A case-control study in the UAE indicated that 90.3% of those with speech and language developmental delays used electronic devices [6]. These findings underscore the need for ongoing monitoring of children's language development and provision of appropriate support services for those at risk of speech delay.

A previous study in Jeddah, Saudi Arabia, showed no relationship between the duration of smart device usage and speech delay (P = 0.538) [25], which is consistent with our study. In addition, a study of preschool children in Makkah revealed that maternal education was one of the most significant risk factors associated with developmental delay [29]. However, our findings contradicted those of several other studies. For instance, a study involving 1,077 children with a median age of 18.4 months reported a significant association between handheld screen time and expressive speech delay [31]. A cohort study in Shanghai, China, found that excessive screen time in early childhood was associated with poor cognitive and social‑emotional development [32]. Furthermore, studies in Finland have reported negative associations between children's lexical and general language abilities and both children's and mothers' screen time [33]. A case-control study in the UAE indicated that early onset of electronic device use (12-24 months) predicted language delays, while factors less likely to be associated with language delays included watching TV and mother's education level [6]. In addition, a cross‑sectional study in Indonesia examining children aged one to two years revealed that more than two hours of daily screen time increased the risk of speech delay by 6.2 times in this age group. Moreover, being male and having low parental education are risk factors for speech delay [34]. A study in Malaysia showed a moderate correlation between higher children's screen time and higher parental screen time, with household income positively correlating with screen time for both children and parents [35].

A review of 12 articles reported that increased screen time and early age of viewing onset had negative effects on language development [36], and a systematic review supported the notion that preschool screen time negatively affects children's cognitive and language development [37]. In addition, a meta‑analysis indicated that screen time increases language and speech delays in children by 2.64 times compared with no screen time, a finding that was statistically significant [38].

The heterogeneous findings across studies indicate the complexity of the relationship between screen time and language development. Multiple factors, including parental education, screen time supervision, and sleep duration, play substantial roles in fostering positive developmental outcomes. Conversely, other studies have illustrated the detrimental effects of excessive screen time, especially during formative years. The variations in these findings suggest that "screen time" is not a uniform exposure. Differences in how screen use is measured (total hours versus specific modalities and content types), the developmental stage at assessment, the degree of parental involvement, and sociodemographic context all likely contribute to variability in observed associations. In the present study, associations with maternal education, supervision, PG familiarity, and sleep duration were more prominent than associations with total daily screen time, implying that the quality and context of media use, together with broader caregiving resources, may be at least as important as cumulative duration for early language outcomes. The discrepancies among these studies further underscore the need for tailored guidelines addressing children's media usage, ensuring that technology serves as a constructive rather than detrimental influence on their developmental environment.

Strengths and limitations

This study has several strengths. It addresses a timely question regarding early childhood screen exposure and language development in a Middle Eastern setting where empirical data remain limited. The inclusion of children across a wide age range, together with parental and household characteristics and screen‑related factors, provides a broader view of the child’s media and caregiving environment. The use of a validated developmental screening tool (A-ASQ‑3 communication domain) strengthens the validity of language outcome measurement.

However, important limitations should be acknowledged. The cross‑sectional design precludes causal inference, and reverse, or bidirectional relationships between screen exposure and language development cannot be excluded. Although an a priori sample size of 377 children was calculated, the final analyzed sample comprised 98 children, which reduces statistical power and limits the ability to detect small‑to‑moderate associations, particularly in subgroup analyses. The wide age range (1-66 months) encompasses multiple developmental stages, and age‑stratified or multivariable analyses were not feasible because of small cell sizes; consequently, associations are unadjusted and should be interpreted as exploratory. Screen exposure variables relied on caregiver self‑report and broad time categories, without detailed information on media modality, language of content, interactivity, or co‑viewing, and parental supervision and PG familiarity were measured with single items. These measurement constraints, together with the modest sample size, may attenuate or obscure dose-response patterns. Finally, the use of non‑random convenience sampling from a single region may limit external generalizability.

Despite these limitations, the study contributes preliminary evidence on how aspects of the home media environment and caregiving context relate to early language development in Saudi Arabia and highlights priorities for more rigorous longitudinal research.

## Conclusions

In this cross‑sectional community‑based study from the Eastern Province of Saudi Arabia, 6.1% of children scored below and 9.2% scored close to the A-ASQ‑3 communication cut‑off, while the majority scored above the cut‑off. Higher communication scores were associated with higher maternal education, closer parental supervision during screen use, longer sleep duration, and caregiver familiarity with PG content ratings, whereas total daily screen time duration alone was not significantly associated with language scores in this sample. These findings suggest that the broader caregiving and media context, including parental monitoring, awareness of age‑appropriate content, and adequate sleep, may be particularly relevant for supporting early language development.

From a practical perspective, counselling of parents and caregivers can emphasize active supervision during children's screen use, selection of age‑appropriate and linguistically rich content, reduction of unnecessary background and late‑evening screen exposure, and promotion of healthy sleep routines, alongside routine monitoring of language development milestones. Larger, longitudinal and multicentre studies that incorporate more detailed exposure measures and developmental mechanisms are needed to clarify temporal relationships, explore dose-response patterns, and inform more nuanced guideline recommendations for early childhood screen use.

## Appendices

Questionnaire

The following appendix tables list the questionnaire items and predefined response options used for data collection. They do not present study results; all analytic results are reported in the main results tables and figures.

Section A

**Table 9:** Questionnaire items and response options: child characteristics A-ASQ-3: Arabic Ages and Stages Questionnaire, third edition

Demographic characteristics		Answer
Age	By months	
sex	Female	
	Male	
Attend kindergarten	Yes	
	No	
Nanny presence at home	Yes	
	No	
living with extended family	Yes	
	No	
Number of children in the family	1 child	
	2-3 children	
	4-5 children	
	More than 5 children	
Birth order		
A-ASQ-3 score		

Section B

**Table 10:** Questionnaire items and response options: parent and household characteristics

Demographic characteristics		Answer
Informant	Father	
	Mother	
	Others	
Marital status	Married	
	Divorced	
Employment status	Both parents employed	
	Father only	
father’s educational	Less than high school	
	High school	
	Diploma/Bachelor	
	Master/PhD	
Mother’s education	Less than high school	
	High school	
	Diploma/Bachelor	
	Master/PhD	
Monthly income (SAR)	Less than 5000	
	5001-10,000	
	10,001-20,000	
	More than 20,000	
Language spoken	Arabic	
	Mix (Arabic and English)	
Interactive communication	less than 1 hour/day	
	1-2 hours/day	
	More than 2 hours/day	

Section C

**Table 11:** Questionnaire items and response options: screen exposure and sleep

Question		Answer
Age of introduction electronic devices	less than 6 months	
	6-12 months	
	between 1-2 years	
	between 2- 3 years	
	More than 3 years	
	Not allowed	
Number screen devices at home	None	
	1-2 devices	
	3-4 devices	
	5-6 devices	
	More than 6	
Does your child own a device	No	
	Yes	
Type of devices	TV	
	Smartphone	
	iPad or tablets	
	Computer or laptop	
	Game console (e.g., PlayStation, Xbox)	
Daily duration of screen time	None	
	Less than 30 mins	
	1-2 hours/day	
	2-3hours/day	
	3-4hours/day	
	More than 4 hours	
Following the parental guidance (PG) score for movies and video games	Yes	
	No	
	Not familiar with PG	
Nature and language of content	Entertainment shows for kids (Cartoons, animation..)	
	Educational (videos, puzzles, reading, or math games..)	
	Games	
	Music	
	Adult content (movies or and TV shows)	
Restriction on screen device time	No	
	Yes	
Duration of restriction	No restriction	
	1 hour/day	
	2 hours/day	
	3 hours/day	
	4 hours/day	
screen exposure during the last 2 hours prior to sleep	Yes	
	No	
Does your child experience sleep problems?	Yes	
	No	
How many hours of sleep your child has	5-9 hours	
	10-14 hours	
	More than 14 hours	
